# Supplementary material for: Blind deconvolution estimation by multi-exponential models and alternated least squares approximations: Free-form and sparse approach
Source: PLoS One. 2021 Mar 18;16(3):e0248301. doi: 10.1371/journal.pone.0248301 (PMC7971520; doi:10.1371/journal.pone.0248301)
Supplement: S1 File — (PDF) [file pone.0248301.s001.pdf]

S1 Acronyms. The following acronyms are used in the document:

ALS	Alternated Least Squares
BDE	Blind Deconvolution Estimation
BDEEL	Blind Deconvolution Estimation by Exponential Library
BDEGME	Blind Deconvolution Estimation by Global Multiple-Exponentials
BDELB	Blind Deconvolution Estimation by Laguerre Basis
BDELME	Blind Deconvolution Estimation by Local Mutiple-Exponentials
DEEL	Deconvolution Estimation by Exponential Library
DEGME	Deconvolution Estimation by Global Multiple-Exponentials
DELB	Deconvolution Estimation by Laguerre Basis
DELME	Deconvolution Estimation by Local Mutiple-Exponentials
FLIM	Fluorescence Lifetime Imaging Microscopy
FluoD	Fluorescence Decay
FluoIR	Fluorescence Impulse Response
FOV	Field of View
FWHM	Full Width at Half Maximum
InstR	Instrument Response
LASSO	Least Absolute Shrinkage and Selection Operator
mFLIM	Multi-Spectral Fluorescence Lifetime Imaging Microscopy
NLS	Nonlinear Least-Squares
NNLS	Non-Negative Least-Squares
PSNR	Peak Signal to Noise Ratio
SNR	Signal to Noise Ratio

■

S2 Appendix: Local FluoIR estimation. This method proposes to jointly estimate the FluoIR parameters $\{\mathbf{c}_k, \boldsymbol{\tau}_k\}$ at the k-th spatial point. For this goal, we make first the following definitions

$$\boldsymbol{\vartheta}_k \triangleq \begin{bmatrix} \mathbf{c}_k \\ \boldsymbol{\tau}_k \end{bmatrix} \in \mathbb{R}^{2N+1}, \tag{45}$$

$$\hat{\mathbf{H}}(\boldsymbol{\vartheta}_k) \triangleq \mathbf{H}^L(\boldsymbol{\tau}_k)\,\mathbf{c}_k \in \mathbb{R}^L, \tag{46}$$

where $\mathbf{H}^L(\boldsymbol{\tau}_k)$ is described in Eq (8), $\mathbf{c}_k$ in Eq (9), and $\boldsymbol{\tau}_k$ in Eq (10). Hence, the optimization problem in Eq (16) is reformulated at the k-th spatial point as

$$\Rightarrow \min_{\boldsymbol{\vartheta}_k \geq 0} \frac{1}{2} \left\| \mathbf{y}_k - \mathbf{U}\,\hat{\mathbf{H}}(\boldsymbol{\vartheta}_k) \right\|_2^2. \tag{47}$$

Given an initial condition $\boldsymbol{\vartheta}_k^0 = \begin{bmatrix} \mathbf{c}_k^0 & \boldsymbol{\tau}_k^0 \end{bmatrix}^\top$, we start an iterative process by index h to estimate the FluoIR parameters in $\boldsymbol{\vartheta}_k$ using a NLS strategy [30, 40]. At the h-iteration, the solution is updated as follows

$$\boldsymbol{\vartheta}_k^{h+1} = \boldsymbol{\vartheta}_k^h + \Delta\boldsymbol{\vartheta}_k^h, \tag{48}$$

where $\Delta\boldsymbol{\vartheta}_k^h \in \mathbb{R}^{2N+1}$ is calculated using a first-order Taylor approximation of the cost function in Eq (47). In this update law, the time constants $\boldsymbol{\tau}_k^{h+1}$ are restricted to a feasible interval $[\tau_{min}, \tau_{max}]$ $(0 < \tau_{min} < \tau_{max})$, and the scalings $\mathbf{c}_k^{h+1}$ are limited to a non-negative condition, i.e. $\mathbf{c}_k \geq 0$. We define the estimated FluoD $\check{\mathbf{y}}_k^{h+1}$ from Eq (5) at the $(h+1)$-iteration as

$$\check{\mathbf{y}}_k^{h+1} \triangleq \mathbf{U}\hat{\mathbf{H}}(\boldsymbol{\vartheta}_k^{h+1}) \approx \underbrace{\mathbf{U}\hat{\mathbf{H}}(\boldsymbol{\vartheta}_k^h)}_{\check{\mathbf{y}}_k^h} + \mathbf{U} \underbrace{\left. \frac{\partial \hat{\mathbf{H}}(\boldsymbol{\vartheta}_k)}{\partial \boldsymbol{\vartheta}_k} \right|_{\boldsymbol{\vartheta}_k = \boldsymbol{\vartheta}_k^h}}_{\boldsymbol{\Phi}_k^h} \Delta\boldsymbol{\vartheta}_k^h, \tag{49}$$

where the Jacobian matrix is given by

$$\boldsymbol{\Phi}_k^h = \begin{bmatrix} 1 & 1 & \cdots & 1 & 0 & \cdots & 0 \\ 1 & e^{\frac{-1}{\tau_{k,1}^h}} & \cdots & e^{\frac{-1}{\tau_{k,N}^h}} & c_{k,1}^h \frac{1}{(\tau_{k,1}^h)^2} e^{\frac{-1}{\tau_{k,1}^h}} & \cdots & c_{k,N}^h \frac{1}{(\tau_{k,N}^h)^2} e^{\frac{-1}{\tau_{k,N}^h}} \\ \vdots & \vdots & \ddots & \vdots & \vdots & \ddots & \vdots \\ 1 & e^{\frac{-(L-1)}{\tau_{k,1}^h}} & \cdots & e^{\frac{-(L-1)}{\tau_{k,N}^h}} & c_{k,1}^h \frac{L-1}{(\tau_{k,1}^h)^2} e^{\frac{-(L-1)}{\tau_{k,1}^h}} & \cdots & c_{k,N}^h \frac{L-1}{(\tau_{k,N}^h)^2} e^{\frac{-(L-1)}{\tau_{k,N}^h}} \end{bmatrix} \in \mathbb{R}^{L \times (2N+1)}$$

Therefore, the estimation error at the $(h+1)$-iteration can be calculated as follows

$$J_k^{h+1} \triangleq \frac{1}{2} \left\| \mathbf{y}_k - \check{\mathbf{y}}_k^{h+1} \right\|_2^2 \approx \underbrace{\frac{1}{2} \left\| \Delta\check{\mathbf{y}}_k^{h+1} - \mathbf{U}\boldsymbol{\Phi}_k^h \Delta\boldsymbol{\vartheta}_k^h \right\|_2^2}_{\Gamma_k^{h+1}},$$

where $\Delta\check{\mathbf{y}}_k^h \triangleq \mathbf{y}_k - \check{\mathbf{y}}_k^h \in \mathbb{R}^L$ represents the estimation error at h-iteration. A similarity metric can be introduced to evaluate the accuracy of the first-order Taylor approximation with respect to the original estimation error, which is defined as

$$\rho_k^{h+1} \triangleq \frac{J_k^h - J_k^{h+1}}{\Gamma_k^h - \Gamma_k^{h+1}}. \tag{50}$$

Following the philosophy of a trust-region optimization [30], a weight matrix $\mathbf{W} > 0$ and a damping term $\lambda^h > 0$ are added into the optimization problem at h-iteration for a Levenberg-Marquardt approach

$$\min_{\Delta\boldsymbol{\vartheta}_k^h} \frac{1}{2} \left\| \mathbf{W^{1/2}} (\Delta\check{\mathbf{y}}_k^{h+1} - \mathbf{U}\boldsymbol{\Phi}_k^h \Delta\boldsymbol{\vartheta}_k^h) \right\|_2^2 + \frac{\lambda^h}{2} (\Delta\boldsymbol{\vartheta}_k^h)^\top \mathbf{R} \Delta\boldsymbol{\vartheta}_k^h, \tag{51}$$

where $\mathbf{R} > 0$. The optimal solution is then given by

$$\Delta\boldsymbol{\vartheta}_k^h = \left(\left[\mathbf{U}\boldsymbol{\Phi}_k^h\right]^\top \mathbf{W}\mathbf{U}\boldsymbol{\Phi}_k^h + \lambda_k^h \mathbf{R} \right)^{-1} \left[\mathbf{U}\boldsymbol{\Phi}_k^h\right]^\top \mathbf{W}\Delta\check{\mathbf{y}}_k^h. \tag{52}$$

As suggested in [40], the damping term $\lambda_k^h > 0$ is updated at every iteration according to the similarity metric ρ_k^{h+1} in Eq (50) to adjust the regularization weight in the least-squares solution of Eq (51). In fact, the update to the linear cost function satisfies

$$\Gamma_k^h - \Gamma_k^{h+1} = \frac{1}{2} \left[\Delta\boldsymbol{\vartheta}_k^h\right]^\top \left\{ \lambda_k^h \Delta\boldsymbol{\vartheta}_k^h + \left[\mathbf{U}\boldsymbol{\Phi}_k^h\right]^\top \mathbf{W}\Delta\hat{\mathbf{y}}_k^h \right\} > 0. \tag{53}$$

As a result, whenever ρ_k^{h+1} is negative or close to zero, the optimal update $\Delta\boldsymbol{\vartheta}_k^h$ in Eq (52) will not improve the original estimation error with respect to $\boldsymbol{\vartheta}_k^h$, so λ_k^h is increased and Eq (52) is recalculated. Otherwise, the damping term λ^h is reduced to avoid restrictions to the update $\Delta\boldsymbol{\vartheta}_k^h$. In this way, we implement an internal search for the damping term λ_k^h in Eq (51) to obtain an improvement in the estimation process inside the NLS methodology.

■

S3 Appendix: Global FluoIR estimation. From the observation model in Eq (6), the FluoIR at k-th spatial point can be written in vector notation $\mathbf{h}_k$ as follows

$$\mathbf{h}_k = \mathbf{H}^G(\boldsymbol{\tau})\,\mathbf{c}_k \quad \forall k \in [0, \hat{K} - 1], \tag{54}$$

where $\mathbf{H}^G(\boldsymbol{\tau})$ is defined in Eq (12), and $\mathbf{c}_k$ in Eq (9). Hence taking advantage of the linear dependence of the scaling coefficients $\mathbf{c}_k$ on the observation model in Eq (6), a LS strategy is followed to compute the optimal components. However, due to the nonlinear dependence of the time constants $\boldsymbol{\tau}$ in (6), a NLS methodology is considered to estimate these parameters over the reduced dataset $\hat{\mathcal{Y}}$ [30, 40].

Therefore, at the $(h+1)$-iteration of the NLS methodology, the previous time constants $\boldsymbol{\tau}^h$ can be used to compute the matrix in Eq (12), i.e. $\mathbf{H}^G(\boldsymbol{\tau}^h)$. Recalling that the scaling coefficients are estimated at k-th available spatial point in the reduced dataset $\hat{\mathcal{Y}}$, a local LS is formulated from Eq (19) as follows

$$\min_{\mathbf{c}_k^{h+1} \geq 0} \frac{1}{2} \left\| \mathbf{y}_k - \mathbf{U}\mathbf{H}^G(\boldsymbol{\tau}^h)\mathbf{c}_k^{h+1} \right\|_2^2, \tag{55}$$

where there are available standard numerical routines to compute this optimization process efficiently [30]. After these $\hat{K}$ local optimization processes, the scaling

coefficients matrix $\mathbf{C}^{h+1} = [\mathbf{c}_1^{h+1} \dots \mathbf{c}_{\hat{K}}^{h+1}] \in \mathbb{R}^{(N+1)\times \hat{K}}$ at h-iteration is constructed. Next, we define an iterative process to estimate the time constants $\boldsymbol{\tau}^{h+1}$ by a NLS strategy over the whole reduced dataset $\hat{\mathcal{Y}}$.

First, we stack all the measurements $\{\mathbf{y}_k\}_{k=1}^{\hat{K}}$ in a large vector $\boldsymbol{\mathcal{Y}}$, and we also define the estimated fluorescence decays $\hat{\boldsymbol{\mathcal{Y}}}$,

$$\boldsymbol{\mathcal{Y}} = \begin{bmatrix} \mathbf{y}_1 \\ \vdots \\ \mathbf{y}_{\hat{K}} \end{bmatrix} \in \mathbb{R}^{L\cdot \hat{K}}, \quad \hat{\boldsymbol{\mathcal{Y}}}(\boldsymbol{\tau}^{h+1}) = \begin{bmatrix} \mathbf{U}\mathbf{H}^G(\boldsymbol{\tau}^{h+1})\mathbf{c}_0^{h+1} \\ \vdots \\ \mathbf{U}\mathbf{H}^G(\boldsymbol{\tau}^{h+1})\mathbf{c}_{\hat{K}-1}^{h+1} \end{bmatrix} \in \mathbb{R}^{L\cdot \hat{K}}, \tag{56}$$

such that the approximation optimization problem becomes

$$\min_{\boldsymbol{\tau}^{h+1}>0} \frac{1}{2} \left\| \boldsymbol{\mathcal{Y}} - \hat{\boldsymbol{\mathcal{Y}}}(\boldsymbol{\tau}^{h+1}) \right\|^2. \tag{57}$$

Under a NLS perspective, the estimated time constants at $(h+1)$-th iteration are given by

$$\boldsymbol{\tau}^{h+1} = \boldsymbol{\tau}^h + \Delta\boldsymbol{\tau}^h, \tag{58}$$

where the update $\Delta\boldsymbol{\tau}^h \in \mathbb{R}^N$ is calculated such that a first-order Taylor approximation of the cost function is minimized. As in the local approach, the updated time constants $\boldsymbol{\tau}^{h+1}$ in Eq (58) are restricted to the feasible interval $[\tau_{min}, \tau_{max}]$. We define the estimated fluorescence measurement $\hat{\boldsymbol{\mathcal{Y}}}$ at $(h+1)$-th iteration as

$$\hat{\boldsymbol{\mathcal{Y}}}^{h+1} \triangleq \begin{bmatrix} \mathbf{U}\mathbf{H}^G(\boldsymbol{\tau}^{h+1})\mathbf{c}_0^{h+1} \\ \vdots \\ \mathbf{U}\mathbf{H}^G(\boldsymbol{\tau}^{h+1})\mathbf{c}_{\hat{K}-1}^{h+1} \end{bmatrix} \tag{59}$$

$$\approx \underbrace{\begin{bmatrix} \mathbf{U}\mathbf{H}^G(\boldsymbol{\tau}^{h})\mathbf{c}_0^{h+1} \\ \vdots \\ \mathbf{U}\mathbf{H}^G(\boldsymbol{\tau}^{h})\mathbf{c}_{\hat{K}-1}^{h+1} \end{bmatrix}}_{\hat{\boldsymbol{\mathcal{Y}}}^h} + \underbrace{\begin{bmatrix} \mathbf{U}\boldsymbol{\Phi}_0^h \\ \vdots \\ \mathbf{U}\boldsymbol{\Phi}_{\hat{K}-1}^h \end{bmatrix}}_{\boldsymbol{\Lambda}^h \in \mathbb{R}^{(L\cdot\hat{K})\times N}} \Delta\boldsymbol{\tau}^h, \tag{60}$$

where the Jacobian matrix is given by

$$\boldsymbol{\Phi}_k^h = \begin{bmatrix} 0 & \cdots & 0 \\ c_{k,1}^{h+1} \frac{1}{(\tau_1^h)^2} e^{-\frac{1}{\tau_1^h}} & \cdots & c_{k,N}^{h+1} \frac{1}{(\tau_N^h)^2} e^{-\frac{1}{\tau_N^h}} \\ \vdots & \ddots & \vdots \\ c_{k,1}^{h+1} \frac{L-1}{(\tau_1^h)^2} e^{-\frac{L-1}{\tau_1^h}} & \cdots & c_{k,N}^{h+1} \frac{L-1}{(\tau_N^h)^2} e^{-\frac{L-1}{\tau_N^h}} \end{bmatrix} \in \mathbb{R}^{L\times N}. \tag{61}$$

As a result, the original estimation error at $(h+1)$-iteration can be approximated as

$$J^{h+1} \triangleq \frac{1}{2} \left\| \boldsymbol{\mathcal{Y}} - \hat{\boldsymbol{\mathcal{Y}}}^{h+1} \right\|_2^2 \approx \underbrace{\frac{1}{2} \left\| \Delta\hat{\boldsymbol{\mathcal{Y}}}^h - \boldsymbol{\Lambda}^h \Delta\boldsymbol{\tau}^h \right\|_2^2}_{\Gamma^{h+1}}, \tag{62}$$

where $\Delta\hat{\boldsymbol{\mathcal{Y}}}^h \triangleq \boldsymbol{\mathcal{Y}} - \hat{\boldsymbol{\mathcal{Y}}}^h \in \mathbb{R}^{L\cdot\hat{K}}$. In this way, a similarity metric can be introduced to evaluate the accuracy of the first-order Taylor approximation with respect to the original estimation error

$$\rho^{h+1} \triangleq \frac{J^h - J^{h+1}}{\Gamma^h - \Gamma^{h+1}}. \tag{63}$$

Furthermore, a matrix weight $\mathbf{W} > 0$ is added into the optimization problem at h-th iteration, as well as, a damping term $\lambda^h > 0$ to reproduce a Levenberg−Marquardt formulation as [30, 40]

$$\min_{\Delta\boldsymbol{\tau}_k^h} \frac{1}{2} \left\| \mathbf{W}^{1/2}(\Delta\hat{\boldsymbol{\mathcal{Y}}}^h - \boldsymbol{\Lambda}^h \Delta\boldsymbol{\tau}^h) \right\|_2^2 + \frac{\lambda^h}{2}(\Delta\boldsymbol{\tau}^h)^\top \mathbf{R} \Delta\boldsymbol{\tau}^h, \tag{64}$$

where $\mathbf{R} > 0$. The optimal solution is then given by

$$\Delta\boldsymbol{\tau}^h = \left(\left[\boldsymbol{\Lambda}^h\right]^\top \mathbf{W}\boldsymbol{\Lambda}^h + \lambda^h \mathbf{R} \right)^{-1} \left[\boldsymbol{\Lambda}^h\right]^\top \mathbf{W}\Delta\hat{\boldsymbol{\mathcal{Y}}}^h. \tag{65}$$

As suggested in [40], and following the philosophy of a trust-region optimization [30], the damping term $\lambda^h > 0$ is updated at every iteration according to the similarity metric ρ^{h+1} to increase or reduce the regularization weight in the least-squares solution of (64). For this purpose, recalling that the actualization in the linear cost function satisfies

$$\Gamma^h - \Gamma^{h+1} = \frac{1}{2}\left[\Delta\boldsymbol{\tau}^h\right]^\top \left\{ \lambda^h \Delta\boldsymbol{\tau}^h + \left[\boldsymbol{\Lambda}^h\right]^\top \mathbf{W}\Delta\hat{\boldsymbol{\mathcal{Y}}}^h \right\} > 0, \tag{66}$$

then if ρ^{h+1} is negative or close to zero, the optimal update $\Delta\boldsymbol{\tau}^h$ in (65) is not improving the original estimation error with respect to $\boldsymbol{\tau}^h$, and λ^h is increased and (65) is recomputed. Otherwise, the damping term λ^h is reduced to avoid limiting the update $\Delta\boldsymbol{\tau}^h$. As a result, we implement an internal search for the damping term λ^h in (64) to always obtain an improvement in the estimation process inside the NLS methodology.

■

S4 Appendix: Instrument response estimation. First, the cost function in Eq (24) is augmented to include the normalization condition $\sum_l \theta_l = 1$ by a Lagrange multiplier $\mu \geq 0$ [30]:

$$\begin{aligned} \hat{J} &= \frac{1}{2}\mathrm{Tr}\left\{ \left(\mathbf{Y} - \sum_{l=0}^{\hat{L}-1} \theta_l \boldsymbol{\Gamma}_l \right)^\top \left(\mathbf{Y} - \sum_{m=0}^{\hat{L}-1} \theta_m \boldsymbol{\Gamma}_m \right) \right\} + \mu \left(\sum_{l=0}^{\hat{L}-1} \theta_l - 1 \right), \\ &= \frac{1}{2}\mathrm{Tr}\left\{ \mathbf{Y}^\top \tilde{\mathbf{Y}} \right\} - \sum_{l=0}^{\hat{L}-1} \theta_l \mathrm{Tr}\left\{ \mathbf{Y}^\top \boldsymbol{\Gamma}_l \right\} + \frac{1}{2}\mathrm{Tr}\left\{ \left(\sum_{l=0}^{\hat{L}-1} \theta_l \boldsymbol{\Gamma}_l^\top \right) \left(\sum_{m=0}^{\hat{L}-1} \theta_m \boldsymbol{\Gamma}_m \right) \right\} + \mu \left(\sum_{l=0}^{\hat{L}-1} \theta_l - 1 \right) \end{aligned}$$

Therefore, the stationary optimality conditions are

$$\frac{\partial \hat{J}}{\partial \theta_m} = 0 \quad \forall m \in [0, \hat{L}-1], \quad \& \quad \frac{\partial \hat{J}}{\partial \mu} = 0, \tag{67}$$

which result in the following set of $\hat{L}+1$ equations

$$\begin{bmatrix} \delta_{0,0} & \dots & \delta_{0,\hat{L}-1} & 1 \\ \vdots & \ddots & \vdots & \vdots \\ \delta_{\hat{L}-1,0} & \dots & \delta_{\hat{L}-1,\hat{L}-1} & 1 \\ 1 & \dots & 1 & 0 \end{bmatrix} \begin{bmatrix} \theta_0 \\ \vdots \\ \theta_{\hat{L}-1} \\ \mu \end{bmatrix} = \begin{bmatrix} b_0 \\ \vdots \\ b_{\hat{L}-1} \\ 1 \end{bmatrix}, \tag{68}$$

where

$$\delta_m, l \triangleq \mathrm{Tr}\left\{ \boldsymbol{\Gamma}_m^\top \boldsymbol{\Gamma}_l \right\} \qquad \forall m, l \in [0, \hat{L}-1], \tag{69}$$

$$b_m \triangleq \mathrm{Tr}\left\{ \mathbf{Y}^\top \boldsymbol{\Gamma}_m \right\}, \tag{70}$$

whose solution provides the optimal parameters $\{\theta_l\}_{l=0}^{\hat{L}-1}$. If for a certain index $\hat{l} \in [0, \hat{L}-1]$, the resulting parameter $\theta_{\hat{l}}$ violates the non-negativity condition, a practical solution inside the iterative process of BDE is to set it to zero (i.e. $\theta_{\hat{l}} = 0$), and re-scale the parameters to keep the normalization condition (i.e. $\sum_l \theta_l = 1$).

■
