# Supplementary material for: Blind deconvolution estimation by multi-exponential models and alternated least squares approximations: Free-form and sparse approach
Source: PLoS One. 2021 Mar 18;16(3):e0248301. doi: 10.1371/journal.pone.0248301 (PMC7971520; doi:10.1371/journal.pone.0248301)
Supplement: Attachment — Submitted filename: Response_to_Reviewers_PONE-D-20-29880.pdf [file pone.0248301.s002.pdf]

Title: Blind deconvolution estimation by multi-exponential models and alternated least squares approximations

Reviewers' comments

(Reviewers' comments are in boldface and the answers are in italic font)
(*Modifications in the paper have been highlighted in **blue color***)
(Line numbers are referred to the revised paper, not to the original one)

First, the authors would like to thank the reviewer and the Editor for their valuable comments. The main contribution on this new version is the inclusion of a deeper critical perspective to the manuscript. We really think that such comments helped to improve the quality of the manuscript.

%%%%%%%%%%%%%%%%%%%%%%

Journal Requirements:

When submitting your revision, we need you to address these additional requirements.

1. Please ensure that your manuscript meets PLOS ONE's style requirements, including those for file naming. The PLOS ONE style templates can be found at

https://journals.plos.org/plosone/s/file?id=wjVg/PLOSOne_formatting_sample_main_body.pdf and https://journals.plos.org/plosone/s/file?id=ba62/PLOSOne_formatting_sample_title_authors_affiliations.pdf

RESPONSE

We have carefully revised the PLOS ONE template and we are following all the style requirements in this new version. For the manuscript, we have used the LaTeX template, so all the styles for the heading and fonts are followed.

2. Thank you for stating the following in your Competing Interests section: 'No'

a. Please complete your Competing Interests on the online submission form to state any Competing Interests. If you have no competing interests, please state "The authors have declared that no competing interests exist.", as detailed online in our guide for authors at http://journals.plos.org/plosone/s/submit-now

b. This information should be included in your cover letter; we will change the online submission form on your behalf.

RESPONSE

We have included in the cover letter for this new submission the statement **"The authors have declared that no competing interests exist."**

Additional Editor Comments:

I agree with the reviewer's comments. Please make sure to address them in the revised version.

RESPONSE

We sincerely thank the Editor for the evaluation of our manuscript. In the following, we have included a precise response to each reviewer comment.

%%%%%%%%%%%%%%%%%%%%%%%%

Reviewer #1: Comments to the Author

This work proposed a new method for solving blind deconvolution estimation (BDE) problem in fluorescence imaging microscopy, based on multi-exponential models and alternating least squares. Blind deconvolution is a well studied problem recently, and the idea of alternating minimization idea is not quite new. The reviewer does not find much technical novelty, but an application to a new problem. The work is well-organized overall, while the presentation can be improved. Below are some comments:

1. The abbreviations for fluorescence impulse response (FIR) and instrument response (IR) might be a bit confusing for readers with signal processing background. In signal processing, FIR and IIR are typically for finite impulse response and infinite impulse response, respectively. The authors should consider how to differ from the traditional abbreviations, otherwise readers might got confused with the meaning of FIR and IR at the first glance.

RESPONSE

We acknowledge this important remark by the reviewer, so we have updated the abbreviations for fluorescence impulse response and instrument response to FluoIR and InstR, respectively to avoid a possible confusion to the reader. For consistency, the abbreviation of fluorescence decay was also updated to FluoD in the whole manuscript (see page 20).

2. There are a couple of recent papers are related and might be cited:

[1] Joshua T Vogelstein, Adam M Packer, Tim A Machado, Tanya Sippy, Baktash Babadi, Rafael Yuste, Liam Paninski, Fast non-negative deconvolution for spike train inference from population calcium imaging

[2] Johannes Friedrich, Pengcheng Zhou, Liam Paninski, Fast online deconvolution of calcium imaging data

[3] Yenson Lau, Qing Qu, Han-wen Kuo, Pengcheng Zhou, Yuqian Zhang, John Wright. Short-and-Sparse Deconvolution – A Geometric Approach, ICLR’20.

In particular, the reviewer finds the proposed method is quite close to those of [1,2], though [1,2] considered a different application in Calcium imaging. In [1,2], the authors assumed some sparsity structures on FIR, while the proposed method does not. Some more comments and detailed

comparison discussions are needed. In particular, the method might be improved by imposing sparsity structure on FIRs similarly, or other structures.

RESPONSE

We thank deeply the reviewer for bringing to our attention these three relevant works [1,2,3] related to blind deconvolution estimation (BDE). We reviewed carefully the three previous contributions and we detected key differences with our BDE formulation. Contrary to [1,2], in our approach, the excitation signal does not have a spike train shape, and our observation model relies on a convolution with a multi-exponential kernel, compared to the autoregressive structure in the references. Furthermore, the main goal in our work is to reconstruct the fluorescence impulse response in each pixel of the FLIM measurement, nor to identify the spike train of the excitation signal, as in [1,2]. In addition, our formulation considers a free-form for the excitation signal, which is common to all pixels in the FLIM image, but with some sparsity condition as well. Hence, for FLIM datasets, the excitation signal is one narrow pulse without repetitions. Meanwhile, the fluorescence impulse responses have a smooth monotonic exponential decay over the whole measurement interval, so we cannot assume for them a sparsity condition, as suggested by the reviewer. With respect to [3], our formulation does not assume a short kernel and a sparse activation map. In addition, our multi-exponential kernel is not restricted to a sphere constraint, as in [3]. Also, the extension in [3] to convolutional dictionary learning considers multiple unknown kernels/motifs, i.e., multiple input signals, which is not consistent with the studied BDE formulation. Nonetheless, references [1,2,3] ([33], [34] and [35] in our manuscript) are described in the introduction and the main differences to our formulation are also discussed there (see page 2).

3. Since the problem is highly nonconvex, can the authors provide some convergence guarantees of the proposed optimization method? Some numerical and even theoretical justification is needed. Additionally, for convolution, the implementation could be faster via FFTs.

RESPONSE

We acknowledge the detailed and precise evaluation of our work by the reviewer. Since in our formulation for FLIM datasets, we assume a narrow pulse without repetitions as excitation, and each measured fluorescence decay will exhibit a sharp increase to its peak value, followed by a monotonic decrease. Moreover, all the measurements are scaled to sum-to-one, and also the instrument response is limited to sum-to-one. As a result, the scaled shift symmetry described in [3] will not hold in our BDE formulation. In addition, at each stage of the alternated least squares (ALS) scheme in the local and global approaches, a quadratic approximation problem is solved by either a non-linear least squares or linear least squares. So, at each stage, the estimation error is reduced or at least maintained. Consequently, convergence is guaranteed in the iterative scheme, but only to a local minimum. For this reason, the initialization based on processing the FLIM dataset is a crucial step in our formulation to obtain meaningful results. We include this discussion in third section, while describing the alternated least squares structure (see page 7).

In this new version, we have added a numerical convergence evaluation of the BDE schemes (BDELME and BDEGME) with synthetic FLIM datasets for different noise levels (SNR,PSNR) $\in \{(10,40),(15,45),(20,50),(25,55)\}$ dB, and orders in the multi-exponential model of the synthetic

impulse response (2nd, 3rd and 4th). The stopping threshold is set to $\epsilon_3 = 1 \times 10^{-3}$. The resulting normalized metric Υ^t in the alternated least squares iterations is described below in Fig. 1, where it is observed that in either BDE scheme, the convergence is always monotonic for any noise combination and model order, and just its final steady-state value depends on the noise level. From Fig. 1, we conclude that the local approach (BDELME) converges slower than the global scheme (BDEGME), which could be intuitively expected, since BDELME has roughly twice the free parameters to optimize. For BDELME, the most drastic reductions are achieved in the first five iterations, and for BDEGME, in the first two. In this way, our previous convergence analysis is validated numerically. This previous analysis and discussion is included in page 12.

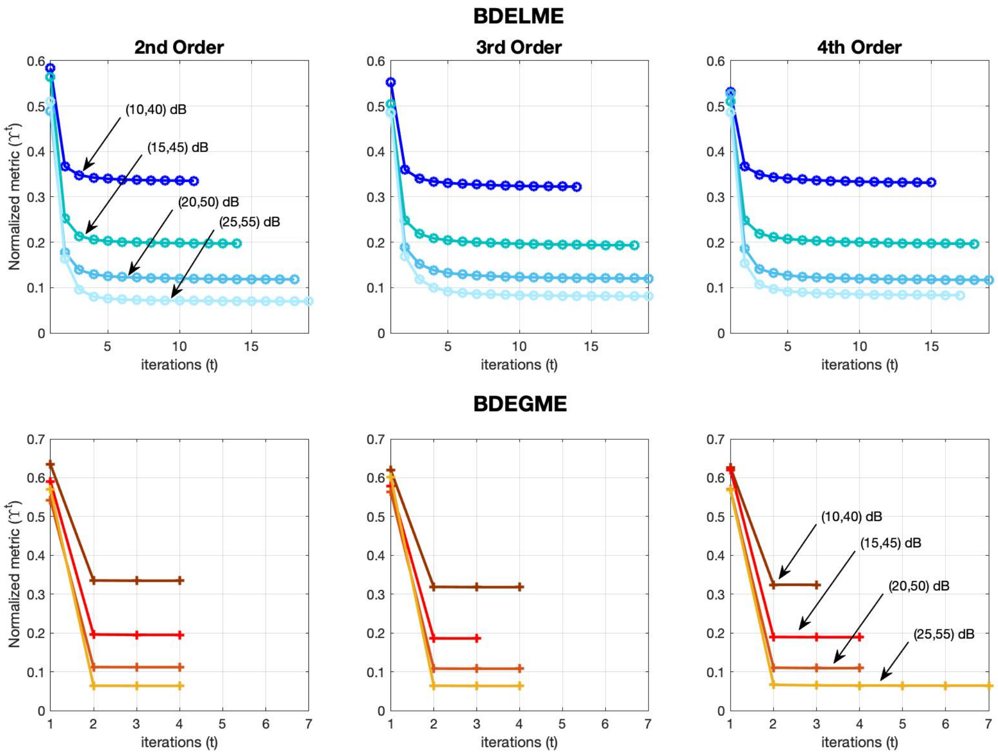

Fig. 1 Normalized metric (Υ^t) vs iteration (t) in the alternated least squares scheme for BDELME and BDEGME by considering synthetic FLIM datasets at different noise levels (SNR,PSNR) ∈ $\{(10,40), (15,45), (20,50), (25,55)\}$ dB, and impulse response orders (2nd, 3rd and 4th).

Now, with respect to the suggestion of implementing the convolution via FFTs, we have chosen to use a time-domain perspective to allow a direct optimization by nonlinear and linear least squares of the parameters in the impulse and instrument responses, nor for the computation of the estimated fluorescence decays. Also, our formulation considers a linear convolution for the observation model. Nonetheless, we thank the reviewer for this interesting suggestion.

4. In numerical comparisons, the authors only compared the previous method by the authors themselves. Comparing with blind deconvolution methods such as those mentioned [1-3], or other generic methods are needed.

We thank this important observation by the reviewer. Nonetheless, the numerical comparison must be under the same mathematical formulation for the deconvolution process. Hence, as we argued in response 2), the performance comparisons with [1], [2], and [3] are not possible, since they address a different estimation problem. In the "Synthetic and experimental validation" section (see pages 10 to 16), we are comparing the performance of our proposed BDE schemes, BDELME and BDEGME, first to two standard deconvolution methodologies that assume known a priori the instrument response: DELME and DEGME (Pelet et al, 2004 & Warren et al., 2013) [17,18]. These two strategies are the common standards in the numerical analysis of FLIM datasets. Next, we use two previous BDE proposals of our same research group: BDELB and BDEEL. In this way, the proposed methodologies, BDELME and BDEGME, are compared against four schemes from the literature, where their advantages are illustrated in synthetic and experimental datasets (see page 11). We hope that the reviewer could identify our effort to compare our proposals, and to evaluate their performance without bias.

[17] Pelet S, Previte M, Laiho L, So P. A fast global fitting algorithm for fluorescence lifetime imaging microscopy based on image segmentation. Biophysical Journal. 2004;87(4):2807–2817. doi:10.1529/biophysj.104.045492.

[18] Warren SC, Margineanu A, Alibhai D, Kelly DJ, Talbot C, Alexandrov Y, et al. Rapid global fitting of large fluorescence lifetime imaging microscopy datasets. PLoS One. 2013;8(8):e70687. doi:10.1371/journal.pone.0070687.
